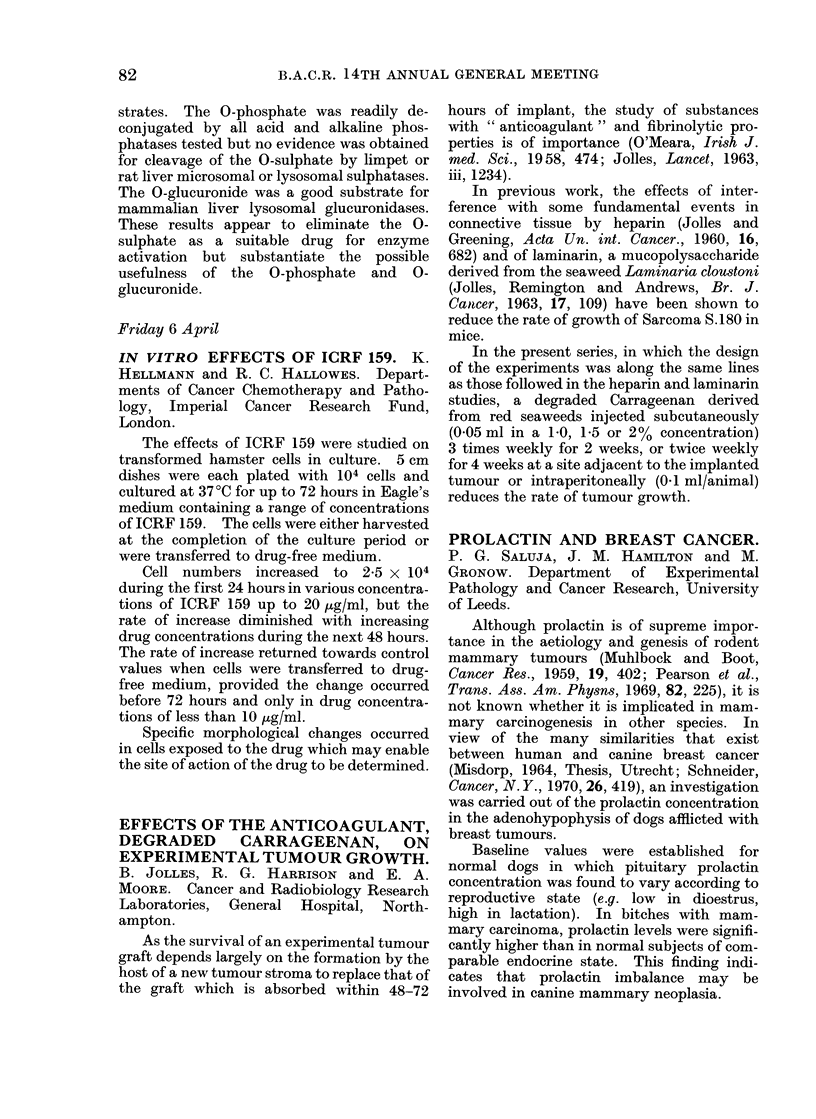# Effects of the anticoagulant, degraded Carrageenan, on experimental tumour growth.

**DOI:** 10.1038/bjc.1973.94

**Published:** 1973-07

**Authors:** B. Jolles, R. G. Harrison, E. A. Moore


**EFFECTS OF THE ANTICOAGULANT, DEGRADED CARRAGEENAN, ON EXPERIMENTAL TUMOUR GROWTH.** B. JOLLES, R. G. HARRISON and E. A. MOORE. Cancer and Radiobiology Research Laboratories, General Hospital, Northampton.

As the survival of an experimental tumour graft depends largely on the formation by the host of a new tumour stroma to replace that of the graft which is absorbed within 48–72 hours of implant, the study of substances with " anticoagulant " and fibrinolytic properties is of importance (O'Meara, *Irish J. med. Sci.*, 1958, 474; Jolles, *Lancet*, 1963, iii, 1234).

In previous work, the effects of interference with some fundamental events in connective tissue by heparin (Jolles and Greening, *Acta Un. int. Cancer.*, 1960, **16**, 682) and of laminarin, a mucopolysaccharide derived from the seaweed *Laminaria cloustoni* (Jolles, Remington and Andrews, *Br. J. Cancer*, 1963, **17**, 109) have been shown to reduce the rate of growth of Sarcoma S.180 in mice.

In the present series, in which the design of the experiments was along the same lines as those followed in the heparin and laminarin studies, a degraded Carrageenan derived from red seaweeds injected subcutaneously (0·05 ml in a 1·0, 1·5 or 2% concentration) 3 times weekly for 2 weeks, or twice weekly for 4 weeks at a site adjacent to the implanted tumour or intraperitoneally (0·1 ml/animal) reduces the rate of tumour growth.